# Epigallocatechin gallate triggers apoptosis by suppressing *de novo* lipogenesis in colorectal carcinoma cells

**DOI:** 10.1002/2211-5463.13391

**Published:** 2022-03-17

**Authors:** Phuriwat Khiewkamrop, Damratsamon Surangkul, Metawee Srikummool, Lysiane Richert, Dumrongsak Pekthong, Supawadee Parhira, Julintorn Somran, Piyarat Srisawang

**Affiliations:** ^1^ Department of Physiology Faculty of Medical Science Naresuan University Phitsanulok Thailand; ^2^ Department of Biochemistry Faculty of Medical Science Naresuan University Phitsanulok Thailand; ^3^ KaLy‐Cell Plobsheim France; ^4^ EA 4267 PEPITE Université de Bourgogne Franche‐Comté Besançon France; ^5^ Department of Pharmacy Practice Faculty of Pharmaceutical Sciences Naresuan University Phitsanulok Thailand; ^6^ Department of Pharmaceutical Technology Faculty of Pharmaceutical Sciences Naresuan University Phitsanulok Thailand; ^7^ Department of Pathology Faculty of Medicine Naresuan University Phitsanulok Thailand

**Keywords:** apoptosis, colorectal cancer, *de novo* lipogenesis, epigallocatechin gallate, PI3K/Akt/mTOR/SREBP‐1c signaling

## Abstract

The *de novo* lipogenesis (DNL) pathway has been identified as a regulator of cancer progression and aggressiveness. Downregulation of key lipogenesis enzymes has been shown to activate apoptosis in cancerous cells. Epigallocatechin gallate (EGCG) inhibits cancer cell proliferation without causing cytotoxicity in healthy cells. The present study aimed to investigate the effects of EGCG on the promotion of apoptosis associated with the DNL pathway inhibition in cancer cells, both *in vitro* and *in vivo*. We observed that two colorectal cancer cell lines (HCT116 and HT‐29) had a higher cytotoxic response to EGCG treatment than hepatocellular carcinoma cells, including HepG2 and HuH‐7. EGCG treatment decreased cell viability and increased mitochondrial damage‐triggered apoptosis in both HCT116 and HT‐29 cancer cells. Additionally, we treated mice transplanted with HCT116 cells with 30 or 50 mg·kg^−1^ EGCG for 7 days to evaluate the apoptotic effects of EGCG treatment in a xenograft mouse model of cancer. We observed a decrease in intracellular fatty acid levels, which suggested that EGCG‐induced apoptosis was associated with a decrease in fatty acid levels in cancer. Suppression of ATP synthesis by EGCG indicated that cell death induction in cancer cells could be mediated by shared components of the DNL and energy metabolism pathways. In addition, EGCG‐induced apoptosis suppressed the expression of the phosphorylation protein kinase B and extracellular signal‐regulated kinase 1/2 signaling proteins in tumors from xenografted mice. Cytotoxic effects in unaffected organs and tissues of the mouse xenograft model were absent upon EGCG treatment.

## Graphical abstract

Epigallocatechin gallate (EGCG) caused cytotoxicity and mitochondrial damage‐induced apoptosis in colorectal HCT116 cancer cells. A decrease in fatty acid levels was associated with EGCG‐induced apoptosis. Additionally, the reduction of ATP generation by EGCG revealed that components of the *de novo* lipogenesis and energy metabolism pathways are implicated in apoptosis. Furthermore, EGCG‐induced apoptosis reduced the expression of phosphorylated protein kinase B and extracellular signal‐regulated kinase 1/2 signaling proteins.

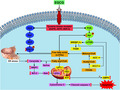

**Abbreviations**
5FU: 5‐fluorouracil
ACC: acetyl‐CoA carboxylase
ACLY: ATP citrate lyase
Akt: v‐akt murine thymoma viral oncogene homolog
AMPK: AMP‐activated protein kinase
ANOVA: analysis of variance
Bcl‐2: B‐cell lymphoma 2
CPT‐1: carnitine palmitoyl transferase‐1
CRC: colorectal cancer
DNL: *de novo* lipogenesis
EGCG: (–)‐epigallocatechin gallate
ERK1/2: extracellular signal‐regulated kinase
FAO: fatty acid oxidation
FASN: fatty acid synthase
HRP: horseradish peroxidase
IC_50_
: inhibitory concentration of 50%
JNK: c‐Jun N‐terminal kinase
MAPK: mitogen‐activated protein kinase
MMP: mitochondrial membrane potential
mTOR: mammalian target of rapamycin
MTT: 3‐(4,5‐dimethylthiazol‐2‐yl)‐2,5‐diphenyltetrazolium bromide
OXPHOS: oxidative phosphorylation
PI3K: phosphoinositide 3 kinase
ROS: reactive oxygen species
SREBP‐1c: sterol regulatory element‐binding protein 1c

Recurrence, metastasis, invasiveness and multi‐drug resistance are all common adverse effects of cancer therapy, and cytotoxic side effects remain a barrier to effective cancer cell treatment. As a result, effective new cancer therapy agents could be used as an alternative to improve the efficiency of clinical research. Colorectal cancer (CRC) is the third greatest cause of death from cancer, with an increasing incidence rate around the world [1]. Surgery and chemotherapy have long been the top choices for CRC patients, but the scarcity of effective treatments has made the prognosis for CRC patients even less promising. Aside from the failure of single‐agent chemotherapy to produce a favorable outcome, the use of multiple‐agent chemotherapy, which includes 5‐fluorouracil (5FU), oxaliplatin and the receptor tyrosine kinase inhibitor sorafenib, is still highly questionable in terms of improved response and prognosis [2]. Several plants are currently being challenged to gain a more efficient treatment and overcome the limitations of traditional cancer therapies in a variety of cancers [3, 4, 5, 6]. As a result, the development of new therapeutic strategies based on the anticancer activity of natural plant extracts has shed light on prospective regimens for circumventing the limitations of chemotherapy and improving treatment outcomes in CRC patients.

The aberrant regulation of metabolic pathways is a specific cancer characteristic; it promotes the over‐proliferation of cancer cells and their resistance to cell death stimulations. Warburg’s hypothesis has been reported in cases where increased glucose uptake elevates glycolysis and activates excess levels of pyruvates to be metabolized to lactate, rather than through mitochondrial oxidative phosphorylation (OXPHOS), without depending on a sufficient oxygen supply [7]. In addition, as a result of the high proliferative signal, intermediates from OXPHOS are delivered to the *de novo* lipogenesis (DNL) pathway where they help provide precursors for ATP generation, cell membrane synthesis and various signaling pathways implicated in cancer growth and metastasis [8].

Fatty acid synthase (FASN), one of the key enzymes in the DNL pathway involved in saturated long chain fatty acid synthesis, is expressed at low levels in normal cells, but increased levels have been reported in various cancer cells. Potential therapeutic effects exerted via suppressing fatty acid synthesis in the DNL pathway could indicate a vital association between the DNL pathway and cancer progression, poor prognosis and therapeutic outcomes [9]. Well‐known FASN inhibitors, including cerulenin, C75 and orlistat, suggest the DNL pathway as an attractive therapeutic target for cancer [10, 11]. Several studies have reported that the expression of FASN is regulated by sterol regulatory element‐binding protein 1c (SREBP‐1c), a downstream signaling pathway of phosphoinositide‐3 kinase (PI3K)/protein kinase B (Akt; also known as PKB)/mammalian target of rapamycin (mTOR) axis, mitogen‐activated protein kinase (MAPK) pathway and hypoxic inducible factor‐1α [12, 13]. Inhibiting the expression of these pathways decreases the expression of FASN, which in turn inhibits cell proliferation and promotes apoptosis, in both *in vitro* and *in vivo* cancer models [12, 14]. Those findings therefore validated the important role of the DNL pathway in operating cancer survival, and its potential role as a targeted therapy for cancer.

ATP production has been discovered to have a crucial role in the apoptosis of many cancer cells. In human bladder cancer T24 and 5637 cells, fangchinoline found in *Stephania tetrandra* exhibited a significant anti‐cancer effect through lowering ATP levels [15]. Following treatment with dihydroergotamine tartrate generated from ergot alkaloids, a considerable reduction in ATP levels was considered to be a mediator of reactive oxygen species (ROS)‐induced apoptosis with mitochondrial damage in human lung cancer cell lines [16]. A decrease in ATP production as a result of a reduction of mitochondrial membrane potential after treatment with *Inonotus obliquus* (Chaga mushroom) polysaccharides resulted in apoptosis in lung cancer cells [17]. In prostate cancer cells and mouse xenograft tumors, apoptosis was resulted from suppression of cellular ATP synthesis as a result of disrupted mitochondrial respiration (oxygen consumption) by alternol isolated from fermentation products of a mutant fungus treatment [18]. ATP influenced cell cycle and proliferative proteins implicated in apoptosis, according to a study on MCF‐7 cells [19]. Based on these findings, ATP is considered to be one of the possible mediators that controls apoptosis in cancer cells.

*Camellia sinensis* or green tea contains around 40% polyphenolic chemicals including catechins, in its dry leaves, with epigallocatechin gallate (EGCG) being the predominant catechins component [20]. Black tea, coffee, berries, grapes and wine all contain catechins. Catechin‐containing foods should be included in a regular diet because they have many health‐promoting properties. Anti‐inflammatory, antioxidant and chemopreventive qualities are the most recognized effects of the catechin group [21]. Flavonoids are the most prevalent polyphenolic secondary compounds derived from plant metabolites found in plant‐based food such as fruits, flowers, vegetables, grains and wine [22]. Flavonoids are composed of flavones, flavonols (3‐hydroxyflavone), flavanols, isoflavones and anthocyanidins, which all have the same flavan backbone structure [23, 24]. Flavonoids have significant antioxidant effects as a result of the presence of many hydroxyl groups in the molecules [21]. Flavan‐3‐ols also known as flavanols are primarily found in catechin epimers [(+)‐catechin and (−)‐epicatechin] [25]. The galloyl moiety of tea catechins plays an important role in biological advantages, with the galloyl moiety of catechins EGCGC having the most biological activity when compared to other tea catechins [26].

EGCG promotes apoptosis and inhibits migration in several cancer cells through various pathways [26, 27, 28]. In bladder cancer cells, these effects may be achieved through inhibiting the expression of matrix metalloproteinase (MMP)‐9 and the PI3K/Akt axis [29]. Treatment of hepatocellular carcinoma cells with EGCG causes an inhibition of the expression and activity of phosphofructokinase and anti‐apoptotic protein B‐cell lymphoma2 (Bcl‐2), which facilitates apoptosis [28]. The suppression of the DNL pathway in many cancer cells has been found to be correlated with an anticancer action of EGCG in promoting apoptosis, with a negative effect on normal cells [27]. By contrast to other fatty acid synthesis inhibitors, catechins from green tea enhance this antitumor effect in *in vivo* models without causing weight loss or anorexia side‐effects [30], suggesting that EGCG is a satisfactory anticancer agent. Furthermore, a positive correlation of lipid content and resistance to chemotherapy and nutritional deprivation will shed some light information on the usage of EGCG in combination with other metabolic stress strategies with the purpose of improving cancer therapeutic efficiency [31].

The present study investigated the effects of EGCG on the inhibition of the DNL pathway, which resulted in apoptosis in colorectal carcinoma HCT116 cells and a tumor xenograft mouse model without causing undesirable toxicity to healthy organs of tumor‐bearing mice. A correlation between apoptosis and fatty acid deprivation that involves ATP synthesis depletion has been identified. EGCG‐induced apoptosis was observed when upstream Akt and extracellular signal‐regulated kinase (ERK)1/2 pathways were depleted. Because EGCG had no adverse effects on the healthy organs of the tumor‐bearing host; it was therefore proposed that the EGCG‐targeted reduction in fatty acid and ATP synthesis may be a safe anticancer treatment approach.

## Materials and methods

### Cell culture

HCT116 (ATCC CCL‐247) and HT‐29 (ATCC HTB‐38) human colorectal carcinoma cell lines were cultured in McCoy’s medium (Corning Inc., Corning, NY, USA) containing 10% fetal bovine serum (Gibco, Thermo Fisher Scientific, Inc., Waltham, MA, USA) and 1% penicillin–streptomycin (Gibco, Thermo Fisher Scientific, Inc.) and incubated at 37 °C in a 5% CO_2_ humidified incubator. In addition, HepG2 (JCRB1054) and HuH‐7 (JCRB0403) human liver cancer cell lines obtained from the Japanese Collection of Research Bioresources Cell Bank (JCRB Cell Bank, National Institutes of Biomedical Innovation, Ibaraki‐shi, Japan) were cultured in Dulbecco’s modified Eagle’s medium (Corning, Inc.) supplemented with 10% fetal bovine serum and 1% penicillin–streptomycin and incubated at 37 °C in a 5% CO_2_ humidified incubator. A normal ATCC SCRC‐1041 human fibroblast foreskin HFF‐1 cell line was cultured in Dulbecco’s modified Eagle’s medium (Corning, Inc.) supplemented with 10% fetal bovine serum and 1% penicillin–streptomycin and incubated at 37 °C in a 5% CO_2_ humidified incubator.

The culture media were replaced every 3 days. Complete growth media were freshly prepared every week. Moreover, 80–90% of cell confluence was subcultured. Cell morphology and numbers were recorded for every subculture, avoiding the use of cells exceeding 10 subculture passages to verify the normal growth of cells and ensure the reliability and consistency of the results. Cultures were detected for mycoplasma contamination by staining DNA with 4′,6‐diamidino‐2‐phenylindole, dihydrochloride (D1306; Molecular Probes, Thermo Fisher Scientific, Inc.) and visualized using a fluorescence microscope (Axio Observer A1; Carl Zeiss AG, Jena, Germany).

### Determination of cell viability using a 3‐(4,5‐dimethylthiazol‐2‐yl)‐2,5‐diphenyltetrazolium bromide (MTT) assay

An MTT (Amresco, LLC, Solon, OH, USA) assay was used to evaluate cancer cell viability following treatment. Cells were plated in 96‐well plates for 24 h. They were then incubated with various concentrations of EGCG (CAS 989‐51‐5; EMD Millipore, Burlington, MA, USA) for 24 h. The vehicle control cells were incubated with 0.1% dimethyl sulfoxide only. After 24 h of treatment, MTT solution (10 mg·mL^−1^) was added to the harvested cells and incubated for 4 h at 37 °C in a 5% CO_2_ humidified incubator. Formazan violet crystals of MTT were dissolved using dimethyl sulfoxide and detected for absorbance at a 595‐nm wavelength using a microplate reader (BioTek Instruments, Inc., Winooski, VT, USA). Cell viability percentages were calculated and compared with the control using prism, version 9 (GraphPad Software Inc., San Diego, CA, USA).

### Evaluation of apoptotic stages by annexin V/propidium iodine (PI) staining

An apoptosis assay was performed using double‐staining dyes, including Alexa Fluor 488 Annexin V and PI; (Thermo Fisher Scientific, Inc.), which conjugate with phosphatidyl serine at the outer membrane of the cells to indicate an early stage of apoptosis, as well as DNA content in the nucleus to indicate a late stage of apoptosis. Cells were seeded in 24‐well plates and incubated overnight. Cells were collected after 24 h of treatment and stained with Alexa Fluor 488 Annexin V and PI at room temperature under light protection. Apoptotic rates were analyzed by FACScalibur flow cytometry using cellquestpro (BD Biosciences, Franklin Lakes, NJ, USA) .

### Investigation of mitochondrial membrane potential by JC‐1 staining

Mitochondrial membrane potential (∆Ψm) damage is the initiator of apoptosis in various cancer cells. Cells were stained using JC‐1 dye (Invitrogen, Thermo Fisher Scientific, Inc.), a cationic mitochondrial membrane potential fluorescence probe. A high polarization state of ∆Ψm presents as positive charges of JC‐1 accumulated in the electronegative interior of the mitochondrial matrix and exhibits red fluorescence emission at 590 nm, whereas the disruption of ∆Ψm presents as a decrease in the dye accumulated in the mitochondrial matrix and an increase in the monomeric form in the cytoplasm, which emits green fluorescence at 530 nm. The decrease in the red/green fluorescence intensity ratio represents a dissipation of ∆Ψm. Cells were cultured in a 24‐well plate and incubated overnight. Cells were harvested after 24 h of treatment. Cells were subjected to 20 µm JC‐1, incubated at 37 °C in a 5% CO_2_ humidified incubator for 45 min, and detected using a FACScalibur flow cytometer by excitation at 488 nm and using an emission 585/42 filter. Data were analyzed using cellquestpro.

### Xenograft nude mouse experiment

All experimental animal procedures complied with the standard research protocol for animal care and use established under the ethical guidelines and policies of the National Research Council of Thailand, which were approved by Naresuan University Animal Care and Use Committee. Male BALB/CAJcl‐NU/NU mice (aged 4–8 weeks) were purchased from Nomura Siam International Co. Ltd (Bangkok, Thailand). Mice were acclimatized and housed in pathogen‐free conditions (sterile food and water) in air‐controlled rooms under a 12 : 12 h light/dark photocycle at the Center for Animal Research, Naresuan University. HCT116 cells at a density of 1 × 10^7^ cells were injected into at the right flank of the mice. Tumor volume was measured every 2 days using the formula: tumor volume = tumor length × width^2^/2. After tumors reached 100–150 mm^3^ within 7 days of injection, mice were randomly divided into four groups, which received the following treatments: (a) 0.1% dimethyl sulfoxide as a vehicle; (b) 30 mg·kg^−1^ body weight EGCG (low dose); (c) 50 mg·kg^−1^ body weight EGCG (high dose); (d) 20 mg·kg^−1^ body weight 5FU (Sigma‐Aldrich, Merck KGaA, Darmstadt, Germany) as a positive control. Vehicles, EGCG and 5FU were administered via an intraperitoneal injection every day for 7 days. Tumor size and body weight were measured every 2 days. At the end of the experiment, all experimental mice received an intraperitoneal injection of barbiturate derivatives; sodium thiopental at 100 mg·kg^−1^ to induce death under the assistance of a veterinarian. Internal organs and tumor were collected. Mice were then immediately confirmed death by decapitation after vital organs were removed (all procedures followed the AVMA Guidelines for the Euthanasia of Animals: 2013 and 2020 Edition) [32, 33, 34]. Tumor weight was measured and percentages of tumor volume, tumor size and body weight were calculated, and were then compared with the control using prism, version 9.

### Measurement of protein level in cell and tumor tissue by immunoblotting

Cells were trypsinized and lysed with lysis buffer (mammalian protein extraction reagent), containing 1% protease inhibitors (Thermo Fisher Scientific, Inc.). Tumor tissues were homogenized in RIPA (50 mm Tris‐HCI, pH 7.5 containing 100 mm sodium chloride, 0.1% SDS, 0.1% Triton‐X, 0.5% sodium deoxycholate and 1 mm EDTA) buffer containing 2% phosphatase inhibitor and 1% proteinase inhibitor cocktail (Thermo Fisher Scientific, Inc.). Protein concentration was measured using BCA assay reagent (Thermo Fisher Scientific, Inc.). Aliquots of 50 µg of lysate were separated via 6–12% SDS/PAGE and transferred to the polyvinylidene fluoride membrane. Next, non‐specific proteins on the membrane were blocked using RAPID‐BLOCK solution (Thermo Fisher Scientific, Inc.). The membrane was then probed with the following specific primary antibodies: rabbit anti‐PI3 kinase, p110α (catalog. no. 09‐481), mouse anti‐ERK1/2, clone 16A6.1 (catalog. no. MABS827), rabbit anti‐phospho‐Erk1/2 (Thr202/Tyr204, Thr185/Tyr187), recombinant clone AW39R (p‐ERK1/2; catalog. no. 05‐797R) and rabbit anti‐ATP‐citrate synthase (ACLY; catalog. no. ABC426) (Merck KGaA) at a dilution of 1 : 1000; rabbit anti‐mTOR (catalog. no. 2972S), rabbit anti‐Akt (catalog. no. 9272S), rabbit anti‐p‐Akt (catalog. no. 9271S), rabbit anti‐acetyl‐coenzyme A carboxylase (ACC; catalog. no. 3662S), rabbit anti‐Bak (catalog. no. 3814) and rabbit anti‐β‐actin (catalog. no. 4970) (Cell Signaling Technology, Inc., Danvers, MA, USA) at a dilution of 1 : 500; rabbit anti‐mTOR (phospho S2448) (catalog. no. ab109268), rabbit anti‐SREBP1 (catalog. no. ab28481), rabbit anti‐FASN (catalog. no. ab99359) and mouse anti‐BNIP3 [ANa40] (catalog. no. ab10433) (Abcam, Cambridge, UK) at a dilution of 1 : 500; and rabbit anti‐phospho‐caspase 3 (Ser150) (catalog. no. PA5‐36746; Thermo Fisher Scientific, Inc.) at a dilution of 1 : 400. Consequently, a specific protein was conjugated with goat anti‐rabbit IgG (H+L) secondary antibody, horseradish peroxidase (HRP) (catalog. no. 65‐6120; Thermo Fisher Scientific, Inc.) or goat anti‐mouse IgG (H+L), superclonal recombinant secondary antibody, HRP (catalog. no. A28177; Thermo Fisher Scientific, Inc.) at a dilution of 1 : 7000. Finally, LuminataTM Forte Western HRP Substrate (Merck KGaA) was added to visualize the protein bands, which were then detected via chemiluminescence western blotting (Chemiluminescence Image Quant LAS 4000; Cytiva, Marlborough, MA, USA). The molecular mass marker of proteins in membrane was identified using the BLUeye prestained protein ladder [1BHC‐PM007‐0500 and 1BHC‐PM001‐0500, Bio‐Helix (GeneDirex), GIBTHAI, Bangkok, Thailand]. Protein bands were calculated as a percentage of the relative expression level of protein/actin, and compared with the control using imagej, version 1.46 (NIH, Bethesda, MD, USA).

### Immunohistochemistry and histological assays

The tumor tissue specimens were fixed in 10% neutral formaldehyde, paraffin embedded and sectioned at a thickness of 2–3 µm. Tumor slides were used for immunohistochemistry in accordance with the standard protocol. Consequently, protein expression in tissue slides was detected by incubation with specific primary antibodies for 4 h at 4 °C overnight and conjugated with goat anti‐rabbit IgG (H+L) secondary antibody, HRP (catalog. no. 65‐6120; Thermo Fisher Scientific, Inc.) or goat anti‐mouse IgG (H+L), superclonal recombinant secondary antibody, HRP (catalog. no. A28177; Thermo Fisher Scientific, Inc.). Finally, the expression levels of proteins were detected using 3,3′‐diaminobenzidine substrate (Dako; Agilent Technologies, Inc., Santa Clara, CA, USA), counterstained with hematoxylin and visualized using a light microscope (BX53; Olympus Corporation, Tokyo, Japam). The cytotoxic effect of EGCG on tumor tissues and internal organs of the xenograft host was investigated using hematoxylin and eosin staining. The morphological changes were visualized under a light microscope.

### Fatty acid assay

The level of free fatty acids was investigated using the Free Fatty Acid Quantification kits (United States Biological, Salem, MA, USA). Cells and tissues were homogenized in 1% Triton‐X in chloroform and centrifuged at 15 000 ***g*** for 10 min. The lower phase of homogenized fatty acid was collected by vacuum‐dried chloroform for 30 min at 60 °C. Next, dried lipids were collected, and the free fatty acid level was detected by a microplate reader at an excitation wavelength of 535 nm and an emission wavelength of 590 nm. The calculated percentages of free fatty acids were compared with the control using prism, version 9.

### ATP assay

ATP levels were detected using ATP Quantification kits (United States Biological). Cells and tumor tissues were extracted in ATP assay buffer and centrifuged at 13 000 ***g*** for 5 min. The supernatant was then harvested and the level of ATP was measured by microplate reader at an excitation wavelength of 535 nm and an emission wavelength of 587 nm. The ATP level was calculated using prism, version 9.

### Statistical analysis

All statistical analyses were conducted using prism, version 9. All data were analyzed using one‐way analysis of variance (ANOVA) with Tukey’s post‐hoc test and are presented as the mean ± SD. The samples were analyzed in at least triplicate and compared with the control. *P* < 0.05 was considered statistically significant.

## Results

### Inhibitory effect of EGCG on cell proliferation of colorectal and hepatocellular carcinoma cells

**Fig. 1:**
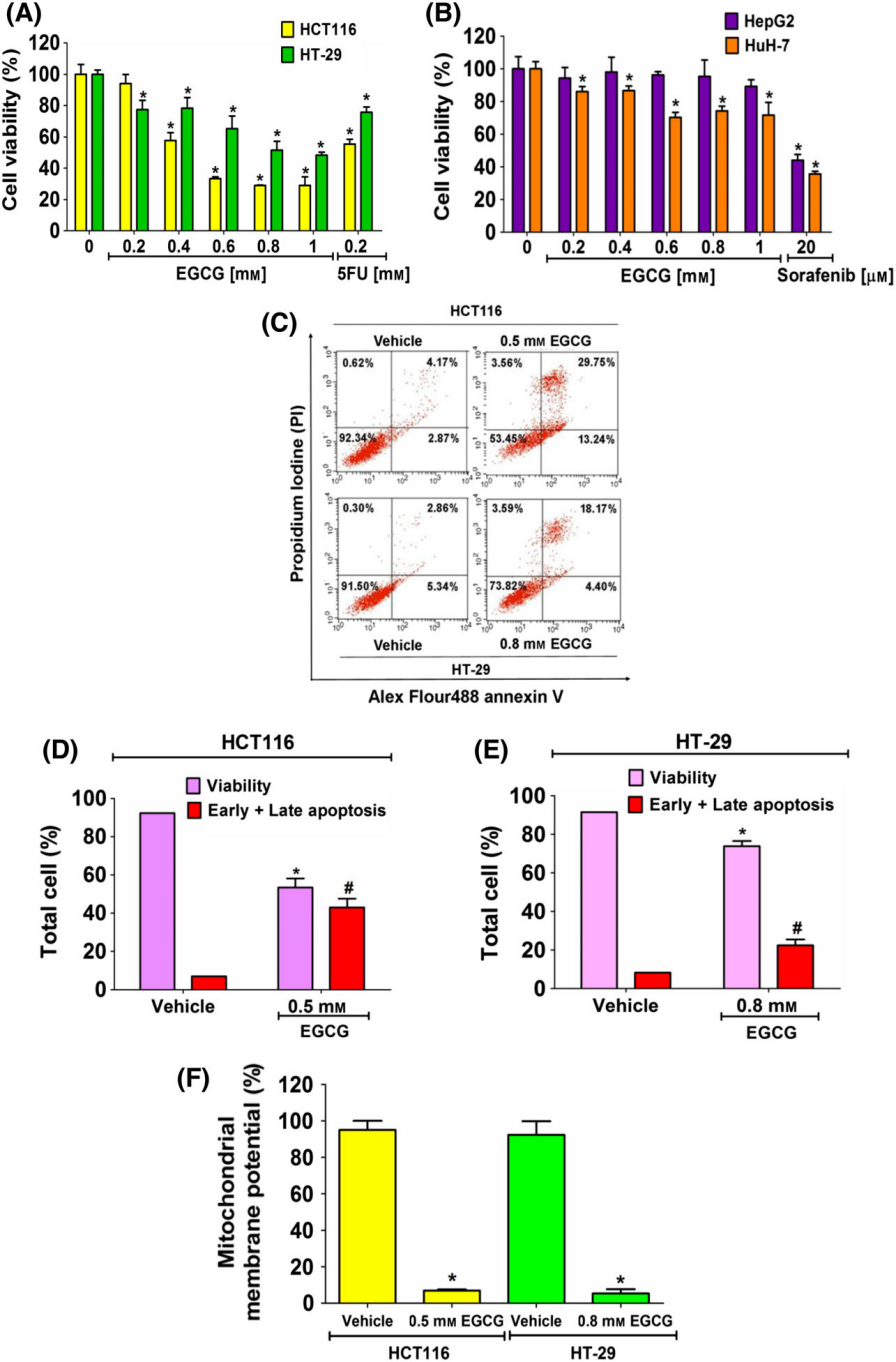
The inhibitory effect of EGCG on cell viability and induction of apoptosis cell death of cancer cells. Cells were incubated with EGCG for 24 h and subjected to a MTT assay. The percentage of cell viability (A) (HCT116 and HT‐29) and (B) (HepG2 and HuH‐7) following treatment was compared with 100% of the vehicle control group. (C) Cells were incubated with EGCG at an IC_50_ concentration for 24 h and then evaluated by annexin V/PI staining. The quantitative bar graphs show percentages of viability and early and late apoptotic cells of (D) HCT116 and (E) HT‐29 cells. (F) The JC‐1 staining assay was used to investigate EGCG‐induced mitochondrial damage. The histograms show the percentages of the red and green fluorescent intensity ratios. Data from at least three independent triplicated experiments are presented as the mean ± SD, *n* = 3. *P < 0.05 and ^#^ *P < *0.05 compared to the control of each cell. All data were analyzed using one‐way ANOVA with Tukey’s post‐hoc test.

Cell lines, including HCT116, HT‐29, HepG2 and HuH‐7, were incubated with EGCG 0‐1 mm for 24 h and cell viability was evaluated by a MTT assay. As shown in Fig. 1, EGCG inhibited cell proliferation in HCT116 and HT‐29 cells in a dose‐dependent manner, at an inhibitory concentration of 50% (IC_50_) of 0.5 ± 0.05 mm and 0.8 ± 0.06 mm, respectively (Fig. 1A). EGCG, on the other hand, had a lower effect on HepG2 and HuH‐7 cells with an IC_50_ of > 1 mm after 24 h of incubation (Fig. 1B). Therefore, CRC cells were more susceptible to EGCG than hepatocellular carcinoma cells. It was also found that EGCG at concentrations of 0.5 and 0.8 mm did not cause any cytotoxicity in HFF‐1 (data not shown), suggesting a safe usage in normal cells. Conventional and effective chemotherapeutic drugs were used as positive controls; 0.2 mm 5FU treatment in HCT116 and HT‐29 CRC cells [35] and 20 µm sorafenib treatment in HepG2 and Huh‐7 liver cancer cells [36] significantly inhibited cell viability when compared to the control cells, which were treated with dimethyl sulfoxide.

### Apoptotic cell death effect of EGCG in CRC cells

Next, the inhibitory effect of EGCG on cancer cell proliferation through triggering apoptosis was evaluated. Following EGCG treatment of HCT116 and HT‐29 cells at IC_50_ values of 0.5 and 0.8 mm for 24 h, respectively, cancer cells underwent early and late stages of apoptosis (Fig. 1C–E). Mitochondrial membrane disruption is one of the central regulations of cytochrome C release into the cytoplasm, which leads to the activation of apoptotic cell death. MMP levels in HCT116 and HT‐29 cells were significantly lower compared to in control cells following 24 h of EGCG treatment, indicating a mitochondria‐dependent apoptotic effect in CRC cells (Fig. 1F).

### EGCG decreases the DNL pathway and ATP level in CRC cells

**Fig. 2:**
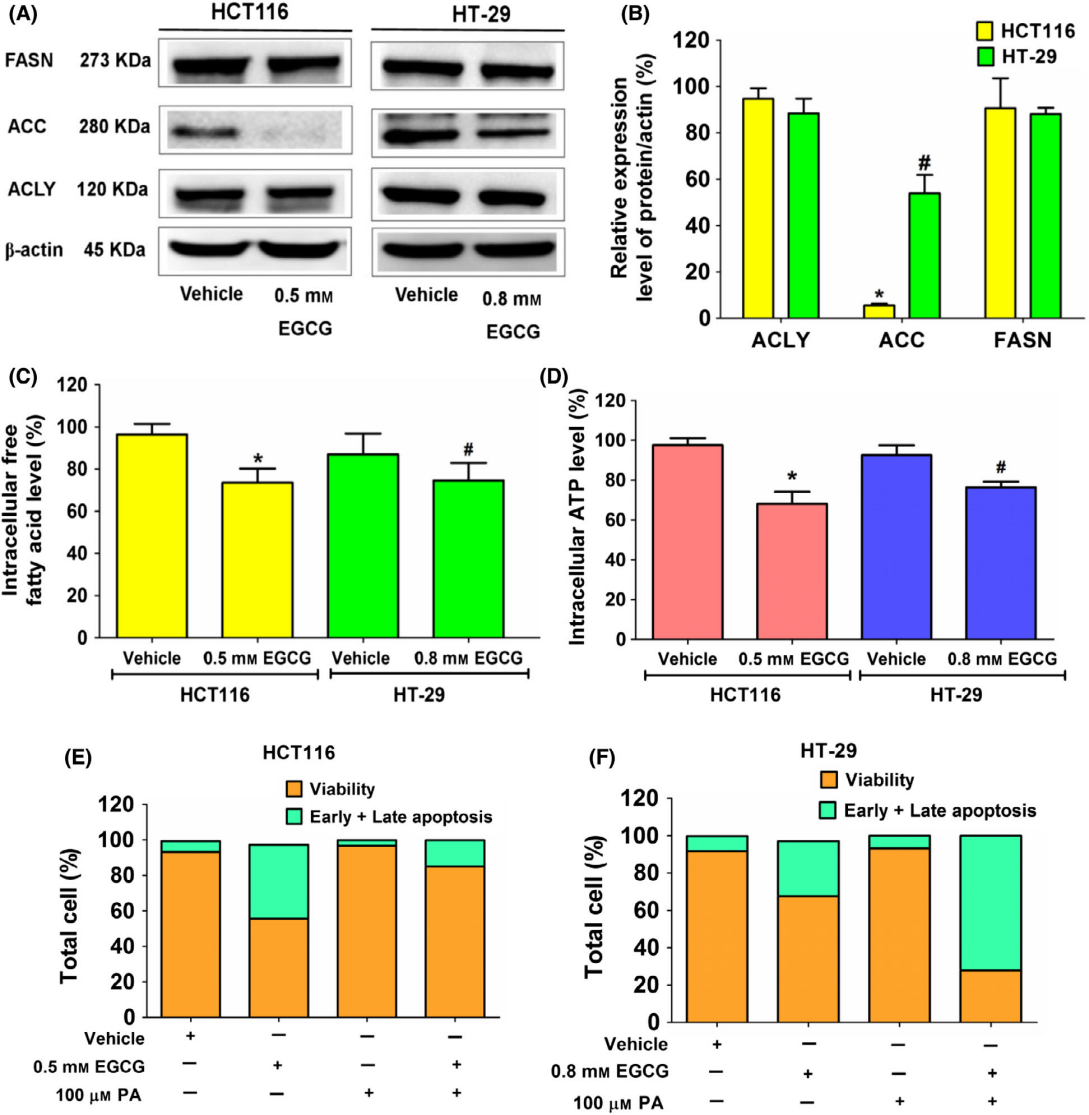
The effect of EGCG on expression of enzymes in the DNL pathway, free fatty acid and ATP levels in colorectal cancer cells, HCT116 and HT‐29 cells. Cells were incubated with EGCG at an IC_50_ concentration for 24 h. (A) An immunoblotting assay was used to assess the expression of enzymes in the DNL pathway. (B) A histogram depicts relative protein expression in comparison to β‐actin. (C) The histogram depicts free fatty acid levels and (D) ATP levels compared to the vehicle control. Apoptotic assay in (E) HCT116 and (F) HT‐29 cells using annexin V/PI staining in EGCG, palmitate (PA) at 100 μm, and a combination of EGCG and PA. Data are expressed as the mean ± SD from at least a triplicate of *n* = 3. *P < 0.05 and ^#^ *P < *0.05 compared to the control of each cell. All data were analyzed using one‐way ANOVA with Tukey’s post‐hoc test.

The downregulation of DNL is considered a potent mediator of apoptosis in cancer cells. The consequent ATP deprivation following DNL suppression plays a role in activating apoptosis [12]. The present study evaluated the expression of the DNL proteins after 24 h of EGCG incubation in HCT116 and HT‐29 cells. The results demonstrated an obvious expression of the DNL proteins in HCT116 and HT‐29 cells, confirming the important role of DNL in CRC metabolism. After 24 h of treatment of HCT116 and HT‐29 cells with 0.5 and 0.8 mm EGCG, respectively, a significant inhibition of the ACC expression was observed in both cell lines. Following EGCG treatment, there was no change in the levels of ACLY and FASN proteins (Fig. 2A,B). In HCT116 and HT‐29 cells, EGCG was showed to significantly inhibit fatty acid production by around 25% (Fig. 2C), suggesting that EGCG has targeted DNL enzyme activity. The inhibition of DNL is controlled by an upstream signaling pathway, AMP‐activated protein kinase (AMPK), which requires ATP/ADP to signal its phosphorylation status [37]. In the present study, EGCG induced a decrease in the ATP level in HCT116 and HT‐29 cells by approximately 30% and 25%, respectively (Fig. 2D). It was therefore suggested that the depletion of lipogenesis in the DNL pathway, which is correlated with ATP suppression, participates in the apoptotic effect of EGCG on CRC cells.

Next, we investigated whether fatty acid depletion was involved in apoptosis following EGCG treatment. Palmitate and other unsaturated long long chain fatty acids produced by the DNL pathway served as cellular signaling networks that controlled cell growth and proliferation via constituting membrane structure and fluidity, and transmitting signals to cascade molecules and protein targets. The depletion of cellular fatty acid disturbs this hallmark architecture that causes cancer cell apoptosis and sensitizes cells to anti‐cancer treatment [38]. Extracellular palmitate supplementation was performed at a concentration of 100 µm, which is equivalent to the physiological fasting concentration in human circulation [39, 40]. However, an excess of palmitate caused cancer cells to deteriorate. Treatment of HepG2 cells with 200 µm exogenous palmitate for 24 h has been reported to elicit oxidative damage in mitochondria, which leads to a decreased activity of all OXPHOS complexes, ultimately resulting in the depletion of ATP expression [41]. It was found that palmitate 100 µm significantly improved the apoptotic activity of EGCG in HCT116 cells, whereas HT‐29 cell apoptosis did not recover following palmitate supplementation (Fig. 2E). Therefore, EGCG‐induced apoptosis may target the inhibition of fatty acid synthesis in HCT116 cells. However, we hypothesized that irreversible and continuous apoptosis induced by EGCG might occur in HT‐29 cells without relying on exogenous palmitate supplementation. Consistent with this hypothesis, a previous study showed that the downregulation of FASN by cisplatin [also known as cis‐diamminedichloroplatinum (II) or CDDP] triggered irreversible DNA damage and continuous apoptosis in MDA‐MB‐231 breast cancer cells, regardless of exogenous fatty acid supplementation [42]. However, further research is needed to fully understand this finding.

### Antiproliferative activity of EGCG and 5FU reduces tumor progression in a nude mouse xenograft model

**Fig. 3:**
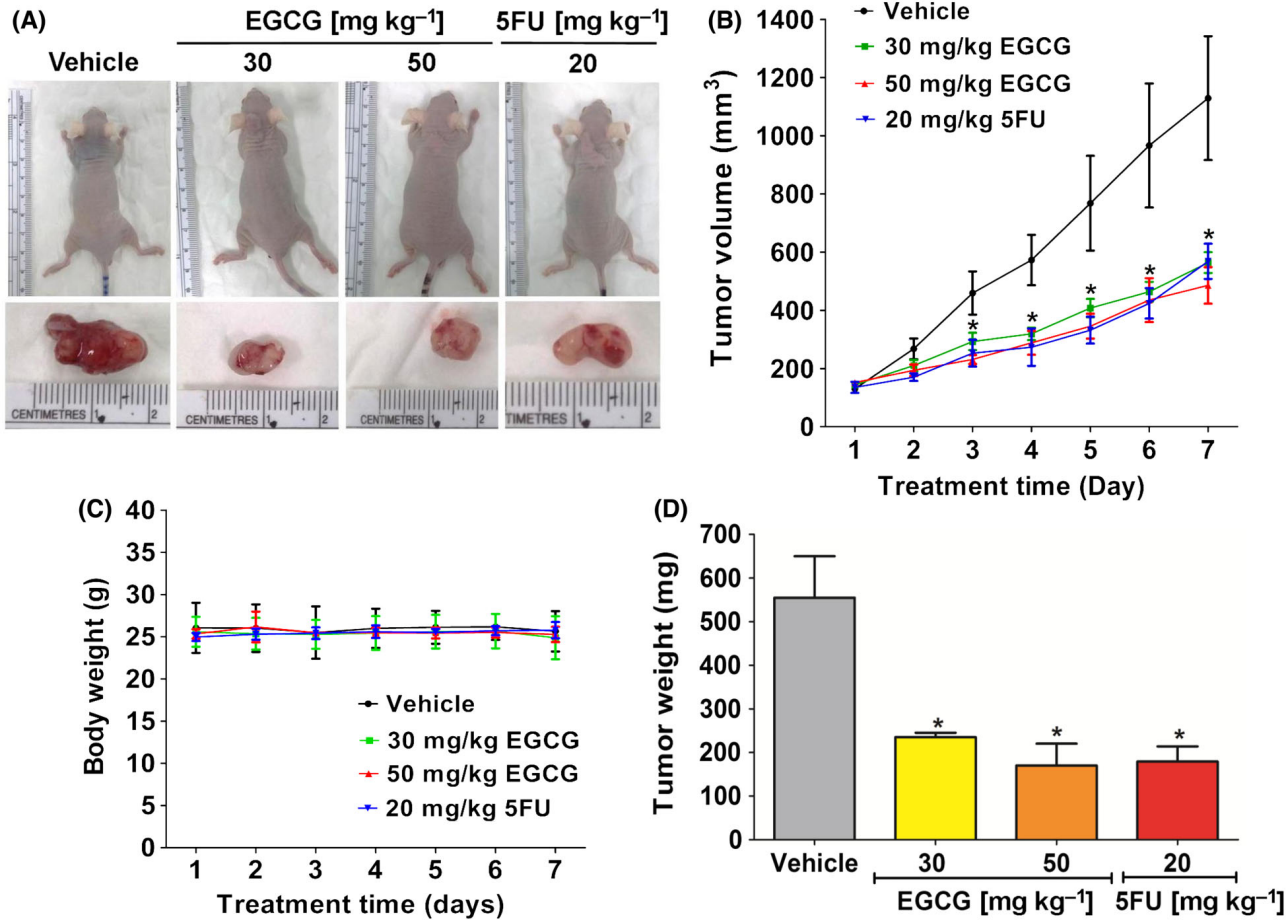
The effect of EGCG on the proliferation of HCT116 tumor xenografts in nude mice. HCT116 cells were injected subcutaneously at the back area of nude mice. (A) Representative images show tumor formation and tumor nodules in nude mice. (B) Tumor volume and (C) body weigh were measured every day for 7 days and (D) tumor weight was measured at the end of the experiment. Representative data used five tumors in each group, presented as the mean ± SD. *P < 0.05 compared to the control. All data were analyzed using one‐way ANOVA with Tukey’s post‐hoc test.

HCT116, which has a lower IC_50_ value than that of HT‐29, was selected for the next evaluation of the apoptotic effects of EGCG on the *in vivo* cancer xenograft mouse model. Treatment with EGCG (30 and 50 mg·kg^−1^ of body weight) via an intraperitoneal injection for 7 days exerted similar level of suppressive effect on the growth rate of HCT116‐tumors by significantly decreasing tumor volume and weight compared to the vehicle control (Fig. 3A,B,D). At the end of the treatment, in the vehicle group, the maximum tumor diameters, including the maximum long and short diameters, were found to be 13 and 18 mm, respectively, with the maximum volume reaching 1521 mm^3^. Of note, the mouse body weight was not influenced by EGCG (Fig. 3C). These results were consistent with those of a previous study reporting that EGCG generates an antitumor effect without cytotoxic effects on body weight loss, as noted in nude mice bearing bladder T24 tumors upon a daily injection of 50 and 100 mg·kg^−1^ EGCG for 28 days [29]. In addition, catechins have been reported to exert an antitumor effect without the side‐effects of weight loss and anorexia, in comparison with other fatty acid synthesis inhibitors [30]. In addition, the present study showed that this antitumor effect of EGCG was similar to that of 5FU at 20 mg·kg^−1^ body weight. 5FU did not cause weight loss compared to the other groups, which was consistent with a previous report [43]. These results demonstrated the consistency of the anticancer effect of EGCG, both in *in vitro* and *in vivo* models, and suggested that EGCG is a safe antitumor agent.

### EGCG increases apoptosis in the tumor xenografts of nude mice

**Fig. 4:**
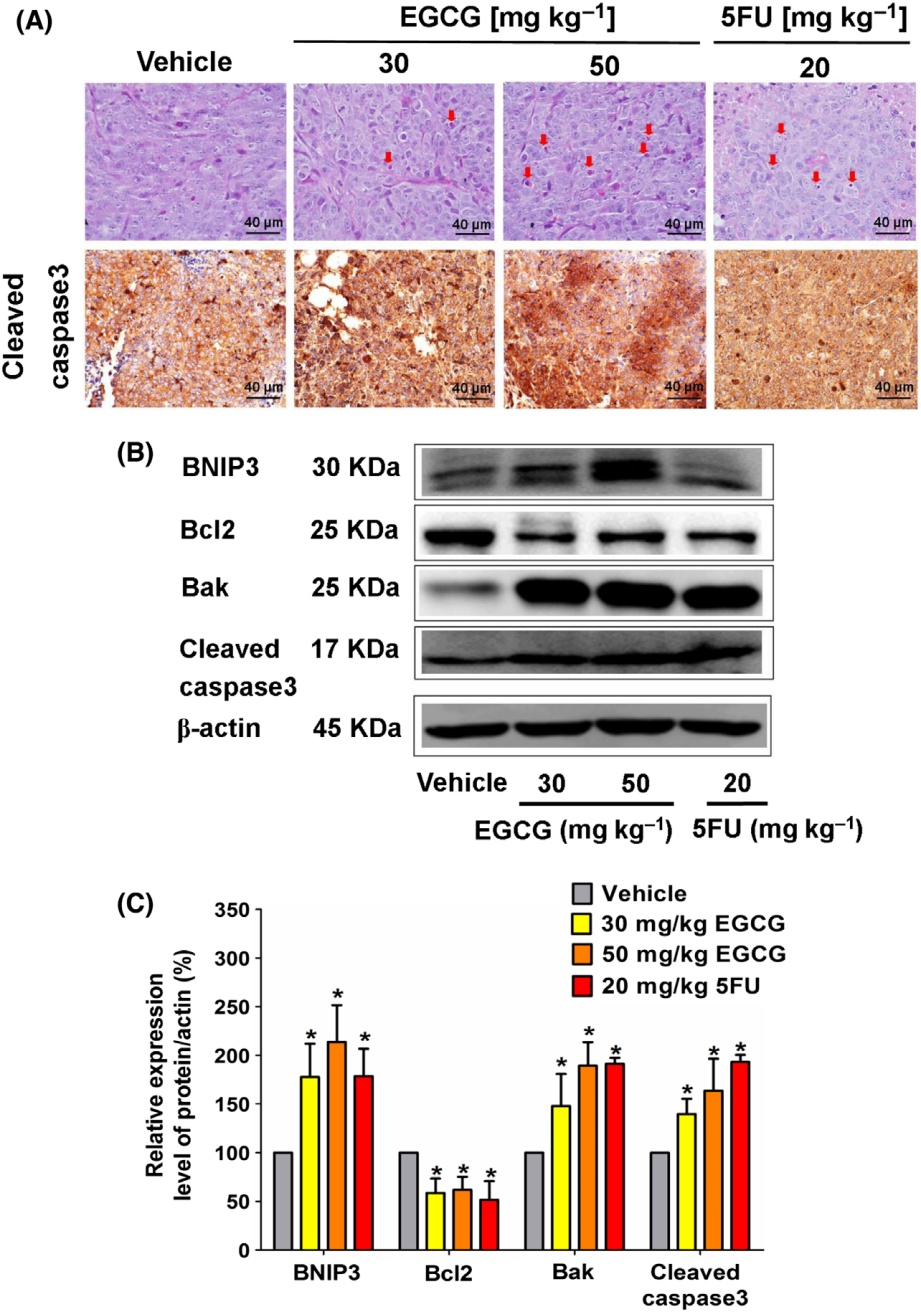
The effect of EGCG on the morphology of the apoptotic characteristics and the expression of proapoptotic proteins in HCT116 tumor xenografts of nude mice. (A) Representative image of tumor hemotoxylin and eosin staining presents the morphology of apoptosis (arrow marks) and immunohistochemistry (scale bar = 40 μm) depicts the intensity of cleaved caspase‐3 expression in tumor tissues following the indicated dose of EGCG treatment. (B) A representative immunoblotting assay shows anti‐apoptotic and proapoptotic protein expression in tumor tissue and (C) a histogram depicts the quantitative protein expression/β‐actin level. Representative data from five tumors in each group were collected and presented as the mean ± SD. *P < 0.05 compared to the control using one‐way ANOVA with Tukey’s post‐hoc analysis.

Next, the apoptosis‐inducing effect of EGCG on mouse tumors was evaluated. Increased DNA breaking and cleaved caspase‐3 protein expression levels were observed in tumors of EGCG‐ and 5FU‐treated mice, with the highest increase being found in the EGCG 50 mg·kg^−1^ body weight group (Fig. 4A). The high‐dose EGCG and 5FU groups significantly enhanced the expression of BNIP3, Bak and cleaved caspase‐3, whereas Bcl‐2 was found to be suppressed (Fig. 4B,C). Although a higher dose of EGCG exhibited a greater apoptotic protein expression level than that in the lower dose group, these results demonstrated the apoptosis induction by EGCG in mouse tumors.

### EGCG decreases the DNL pathway and ATP level in the tumor xenografts of nude mice

**Fig. 5:**
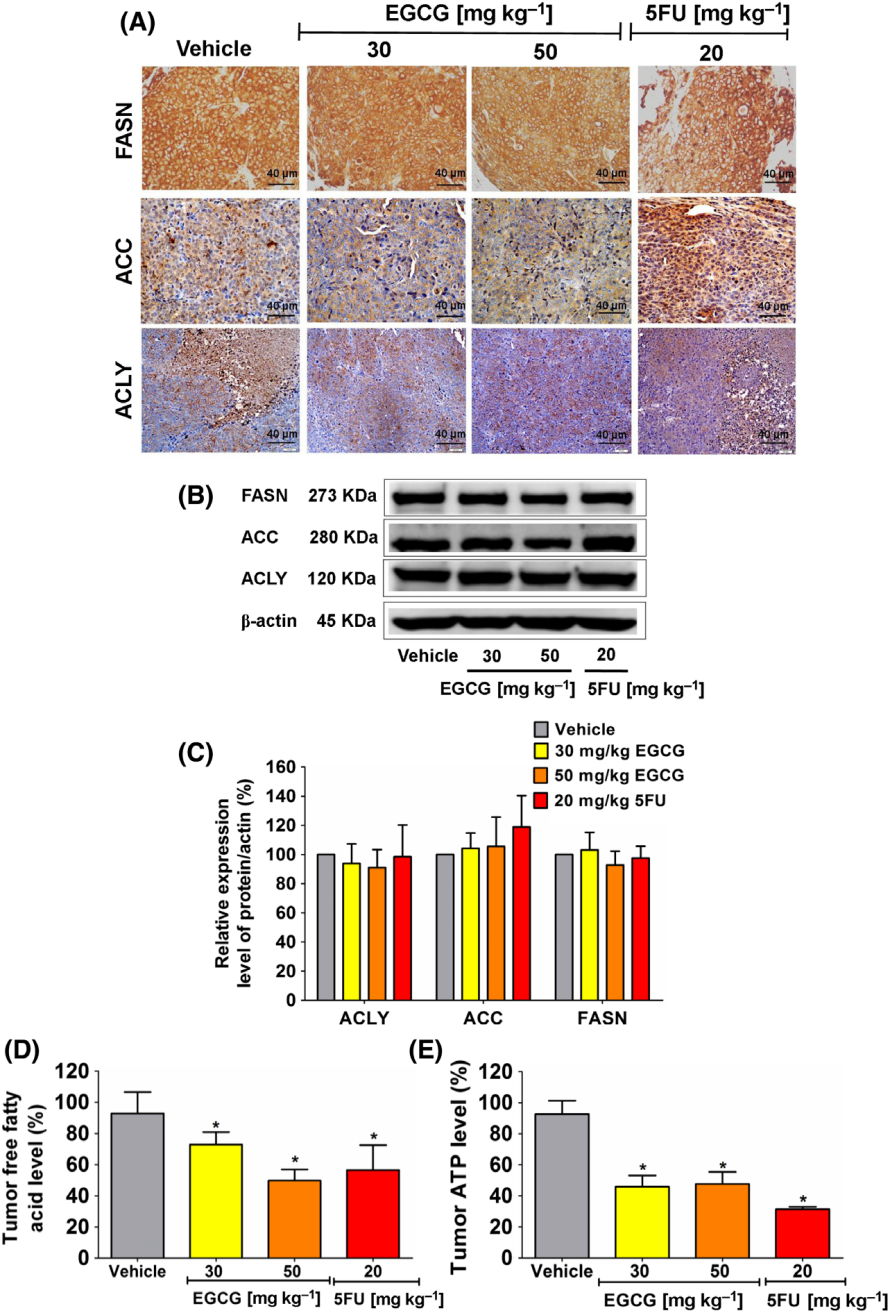
The effect of EGCG on enzyme expression in the DNL pathway, free fatty acid and ATP levels in HCT116 tumor xenografts of nude mice. (A) Representative image of tumor immunohistochemistry staining (scale bar = 40 μm) shows the expression of enzymes in the DNL pathway. (B) ACLY, ACC and FASN expression was measured using an immunoblotting assay and (C) quantitated in a histogram compared to 100% of the control group. (D) The histogram represents free fatty acid and (E) ATP levels. Representative data were collected from five tumors in each group and presented as the mean ± SD. *P < 0.05 compared to the control using one‐way ANOVA with Tukey’s post‐hoc analysis.

Based on the *in vitro* results, a decrease in the DNL pathway potentially enhanced apoptosis in tumors. These *in vivo* tumor data supported the *in vitro* findings. A high concentration of EGCG treatment decreased tumor free fatty acid levels without changing the protein levels in the DNL pathway (Fig. 5A–D). In addition, the reduction in ATP levels was observed in tumors from the EGCG‐treated groups (Fig. 5E). Therefore, in agreement with the *in vitro* findings, lipogenesis depletion in the DNL pathway was correlated with ATP suppression, contributing to the apoptotic effect of EGCG in HCT116 xenograft mouse tumors.

### EGCG decreases the expression of signaling pathway proteins regulating the DNL pathway in the tumor xenografts of nude mice

**Fig. 6:**
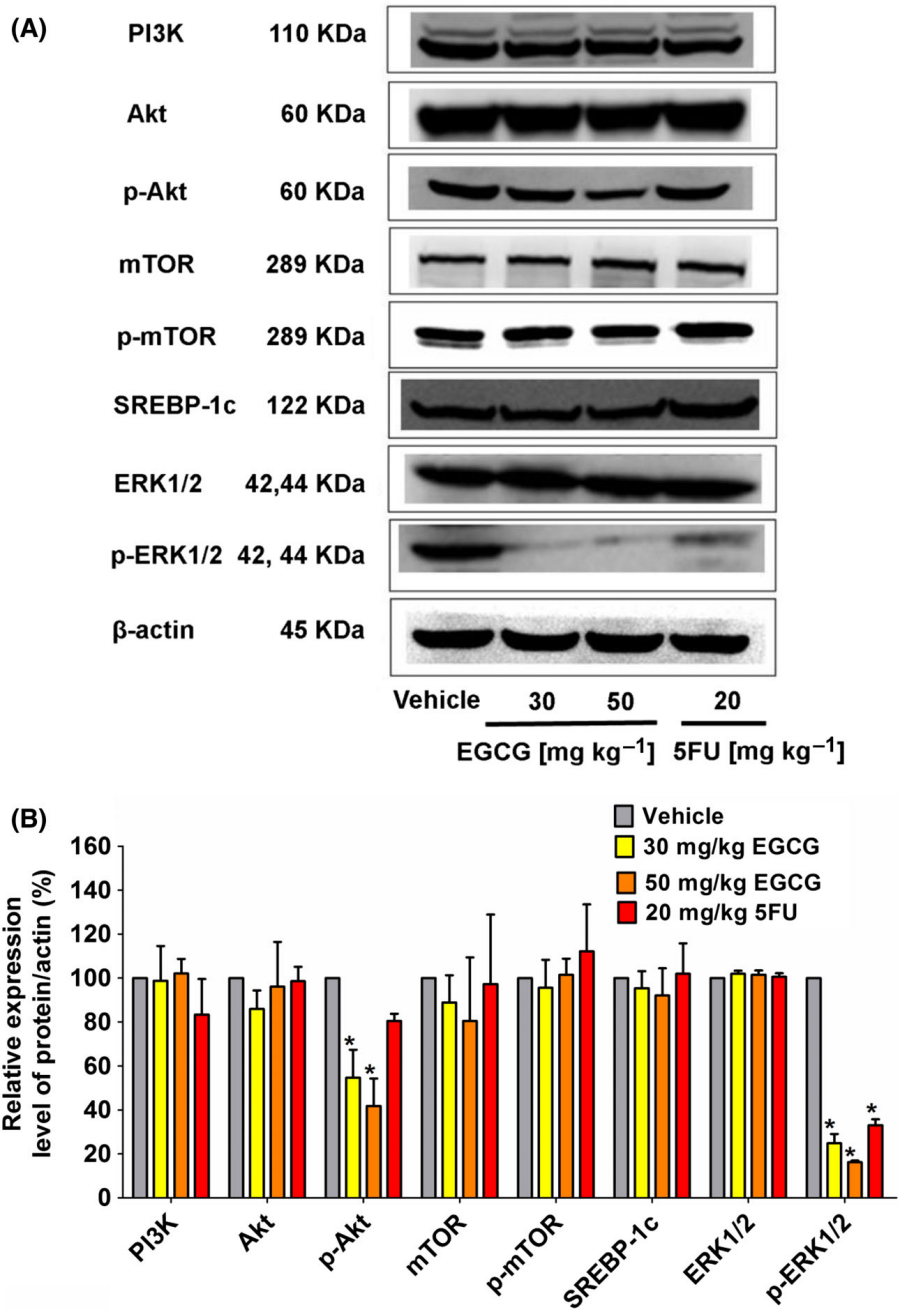
The effect of EGCG on the upstream signaling PI3K/Akt/mTOR/SREBP‐1c and ERK pathways in HCT116 tumor xenografts of nude mice. (A) Representative immunoblotting shows expression proteins collected from five tumors in each group, which was quantitated in a histogram (B) compared to 100% of the control group. The values are presented as the mean ± SD. *P < 0.05 compared to the control using one‐way ANOVA with Tukey’s post‐hoc analysis.

The expression of enzymes in the DNL pathway is generally regulated by the PI3K/Akt/mTOR pathway‐induced modulation of the SREBP‐1c and ERK signaling pathways [44]. In the present study, EGCG treatment was found to reduce the expression of p‐Akt and p‐ERK1/2, whereas 5FU only reduced p‐ERK1/2 expression (Fig. 6). No alteration in the expression of other proteins was observed following EGCG treatment.

### EGCG has no cytotoxic effect on internal organs in HCT116 tumor xenograft‐bearing mice

**Fig. 7:**
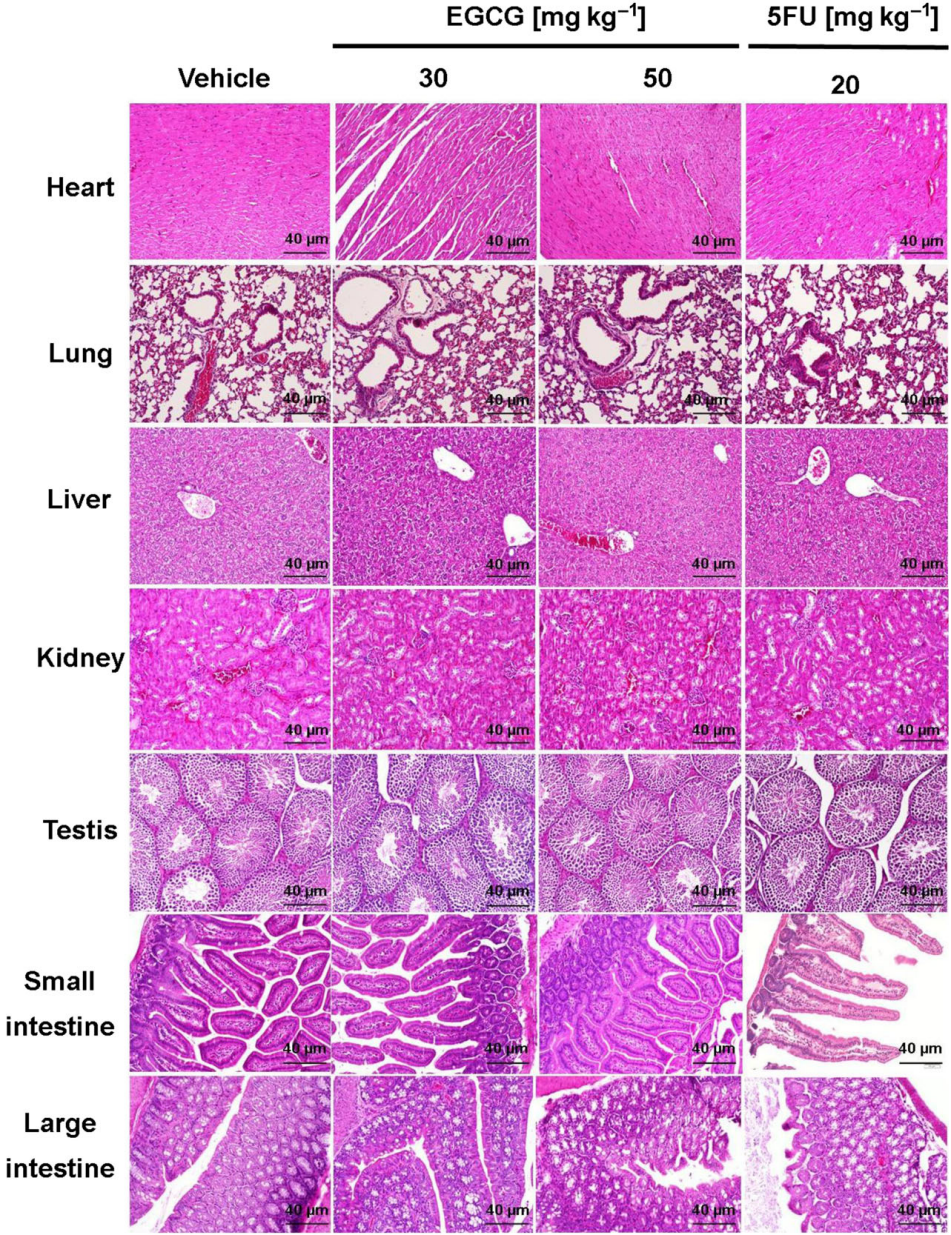
The effect of EGCG on the internal organs of HCT116 tumor xenograft‐bearing mice. Representative images of hemotoxylin and eosin staining from five tumors in each group demonstrate the morphology of internal organs (scale bar = 40 μm) following treatment with the indicated agent and the positive control 5FU.

Finally, the histology of internal organs from each group showed no cellular damage or morphological changes following EGCG and 5FU treatment for 7 days (Fig. 7). Therefore, despite the lack of body weight loss, EGCG treatment was found to have a safe and effective antitumor effect on the tumor‐bearing host.

## Discussion

EGCG is the most abundant polyphenolic compound in dried green tea leaf extracts. Previous studies have reported that EGCG inhibits cell proliferation, metastasis and invasion, and also promotes apoptosis in various cancer cell types [27, 29, 45, 46]. Another study revealed the anti‐metastatic effect of EGCG in the liver metastasis of human CRC [47]. Furthermore, EGCG has been recognized to cause chemosensitivity to the chemotherapy agent 5FU [48], and radiosensitivity to the radiotherapy agent 2‐Gy in CRC cells [49]. In addition, EGCG has been shown to enhance the nasopharyngeal carcinoma response to radiation therapy via the downregulation of FASN expression [50]. In the present study, it was found that EGCG diminished cell viability and increased mitochondrial damage‐induced apoptosis in CRC cells. A reduction of fatty acid synthesis via the DNL pathway was hypothesized to contribute to apoptosis in these cancer cells. In addition, the reduction of fatty acid‐induced apoptosis was associated with a reduced ATP level, both *in vitro* and *in vivo*. The upstream signaling regulation of the DNL pathway‐induced apoptosis, p‐Akt and p‐ERK1/2 expression levels was suppressed by EGCG treatment in mouse tumors.

An abnormally high expression of enzymes in the lipogenesis pathway is one of the most important metabolic features of malignant cancer cells [36, 50]. In the 1920s, Otto Warburg and colleagues observed that tumors absorbed vast amounts of glucose for fermentation to make lactate when oxygen was available; consequently, the name aerobic glycolysis was coined. In 1929, Herbert Crabtree expanded on Warburg's work, discovering and validating Warburg's conclusions [51]. By contrast to normal cells, cancer cells have a preferential shift of citrate intermediates from OXPHOS toward fatty acid metabolism [52]. One of the most abundant saturated fatty acids, palmitate, is required for intracellular metabolism and survival of cancer cells. Palmitate contributes membrane architectures, particularly lipid rafts, which facilitate cellular signaling transduction to support uncontrolled high cell growth rates. The disruption of lipid rafts by FASN inhibition impairs signal transduction pathways, leading to the promotion of cancer cell apoptosis [38]. Therefore, targeting specific metabolic pathways, particularly *de novo* fatty acid synthesis, has been shown to have an efficacy in cancer treatment [53, 54, 55].

Many research groups have reported different concentrations of EGCG that effectively generates cytotoxic effect in HCT116 and HT‐29 cells. However, the clear intensive mechanism has not yet been identified. It has been reported by La *et al*. [48] that the IC_50_ of EGCG treatment for 24 h in HCT116 cells was approximately 270 µm. Morris *et al*. [56] showed the IC_50_ of EGCG treatment for 48 h in HCT116 cells was greater than 100 µm. The EGCG maximum dose at 150 µm for 48 h was further investigated in cell cycle arrest. EGCG treatment at 200 µm for 48 h in HT‐29 cells caused approximately 80% cell death [57]. Haratifar *et al*. [58] reported that incubating HT‐29 cancer cells with EGCG concentrations of 0.4–0.75 mg·mL^−1^ for 24 h resulted in a 50% or greater reduction in cell survival. Despite the fact that EGCG has been shown to have strong anti‐cancer activity in many types of cancer, several animal and epidemiological experimental studies have demonstrated that a single high dose of EGCG (1500 mg·kg^−1^) causes hepatotoxicity and associated dyslipidemia in a dose‐ and route‐dependent manner [59]. Doses of EGCG higher than 750 mg·kg^−1^ trigger several adverse effects in human [60]. In most animal model studies, the doses of EGCG used were below 100 mg·kg^−1^. Maruyama *et al*. [47] used EGCG at 30 mg·kg^−1^ body weight by i.p. injection every other day over a 2‐week period for the treatment in liver metastases. Luo *et al*. [61] reported on a EGCG low‐dose group (25 mg·kg^−1^ EGCG, i.p. injected every day), EGCG medium‐dose group (50 mg·kg^−1^ EGCG, i.p. injected every day) and EGCG high‐dose (EGCG‐H) group (100 mg·kg^−1^ EGCG, i.p. injected every day). Kang *et al*. [62] divided mice into three equal groups (control, 25 mg·kg^−1^ EGCG and 50 mg·kg^−1^ EGCG). Thus, the dose of EGCG that we used in the animal study was based on these previous studies.

The present study demonstrated that the rescue effect of palmitate on the EGCG‐induced suppression of fatty acid synthesis confirms that fatty acids control HCT116 cancer cell proliferation. Exogenous fatty acids have been reported to help rescue the cytotoxic effect of fatty acid synthesis inhibition in a variety of cancer cell types. According to one study, the addition of 100 μm palmitate completely rescued the FASN inhibition‐induced apoptotic effect after α‐mangostin treatment in 3T3‐L1 preadipocytes [63]. Palmitate reversed the inhibitory effect of conjugated linoleic acid on cell growth [64], as well as the cytotoxic effect of amentoflavone in breast cancer cells [65]. It has also been demonstrated that the cytotoxic effect of orlistat involves the inhibition of fatty acid synthesis in PC‐3 cells, with palmitate supplementation being able to reverse this effect [66]. However, in the present study, unlike the results observed in HCT116 cells, exogenous fatty acid supplementation could not overcome EGCG‐induced apoptosis in HT‐29 cells. It could be speculated that HT‐29 cells might be subjected to an irreversible apoptotic process by EGCG, which palmitate could not reverse. Our hypothesis was supported by previous studies, which reported that exogenous fatty acid supplementation did not prevent irreversible DNA damage and continuous apoptosis caused by cisplatin‐induced downregulation of FASN in MDA‐MB‐231 breast cancer cells. [42]. Hopperton *et al*. [52] found no reduction in cytotoxicity after palmitate supplementation in MCF‐7 human breast cancer cells treated with the fatty acid synthesis inhibitors 5‐tetradecyloxy‐2‐furoic acid, C75, or triclosan. Indeed, exogenous palmitate was found to have no effect on glucose deficiency‐induced cell death in hepatoma cells, suggesting that the *de novo* fatty acid biosynthesis is more important for cell proliferation than the fatty acid uptake from the circulation [31]. However, further repeatable experiments in additional cancer cell types are required to examine this hypothesis.

Several agents that target specific metabolic pathways, including fatty acid synthesis, have been shown to have the therapeutic potential to improve cancer treatment efficacy. A reduction of key enzymes in the DNL pathway has been shown to cause a reduction in fatty acid products [27] and fatty acid oxidation (FAO) [12], resulting in a decrease in the proliferative, metastatic and invasive ability of cancer cells [67, 68, 69, 70, 71, 72, 73, 74, 75, 76]. The mechanism of the anticancer effect of EGCG has been demonstrated to be mediated via inhibiting the fatty acid biosynthesis pathway [50, 77, 78, 79, 80]. EGCG inhibits FASN activity, which promotes apoptosis in cancer cells, in the same way that FASN inhibitor C75 does, but with less negative effects [81].

The current *in vitro* experiments revealed that EGCG induced apoptosis by decreasing ACC expression without changing FASN levels. ACC is the rate‐limiting enzyme in the DNL pathway. A decrease in fatty acid synthesis occurs when the ACC is suppressed, which promotes apoptosis. The phosphorylation of ACC by AMPK is essential for inhibiting the DNL pathway [74, 82, 83, 84, 85]. Increased AMPK levels were found to inhibit ACC expression and activity, resulting in a decrease in fatty acid synthesis with no change in FASN protein expression, eventually causing cytotoxicity in SKOV3 human ovarian cancer cells after C93 treatment. It is hypothesized that an increased AMPK/ATP ratio is important in regulating fatty acid production and cell death [86]. ND‐646 inhibited ACC activation in non‐small‐cell lung cancer by binding to the BC domain of ACC and preventing ACC phosphorylation by AMPK [87]. A similar result showed that ACC suppression induced apoptosis in human pancreatic cancer cells treated with CPI‐613 or devimistat, a lipoic acid derivative, but FASN expression remained constant [88]. Malonyl‐CoA substrates for fatty acid synthesis were depleted when ACC was suppressed, resulting in cell death similar to that seen when FASN expression was inhibited [89, 90]. Cells treated with small interfering RNA‐targeting ACC had no effect on the levels of FASN expression. However, after ACC and FASN suppression, cytotoxicity was identical, which was related to oxidative stress and mitochondrial dysfunction [89]. The apoptotic activity of EGCG in breast cancer cells was independent of FASN protein expression levels, although EGCG‐induced apoptosis was demonstrated by downstream proteins AKT and ERK1/2 [91]. Our findings suggested that the inhibition of ACC by EGCG signaling in cancer cells could be linked to AMPK activation.

Although TVB‐3166 is a highly selective FASN inhibitor, it fails to suppress FASN expression in tumor cell line xenografts, and ACC phosphorylation is most likely one of the key causes [38]. In A549 human lung cancer cells, it was observed that apoptotic activity of EGCG‐treated cells was driven by a decrease in FASN activity rather than a decrease in FASN protein levels. By contrast, total levels of FASN protein expression had no effect on the anti‐tumor activity of EGCGC in xenografts [81]. The anticancer effect of FASN activity has been reported to be independent of its expression level [92]. The apoptosis produced by a thiopheno‐pyrimidine‐based FASN inhibitor (Fasnall) in breast cancer cell lines was a result of malonyl‐CoA accumulation, which is known to decrease carnitine palmitoyl transferase‐1 (CPT‐1) activity but had no effect on FASN expression [93]. Following metformin treatment, phosphorylation of ACC significantly increases acetylation levels of histones H3 and H4, as well as other proteins, including α‐tubulin and p65NFκB, which regulate the expression of genes involved in cell development and apoptosis in prostate cancer PC3 and ovarian cancer cells [94]. In HepG2 and LnCap cancer cells, inhibition of ACC by soraphen A triggered apoptotic cell death by suppressing malonyl CoA supply for DNL fatty acid elongation, long chain saturated, monounsaturated and polyunsaturated fatty acid synthesis [95].

It has been proposed that the increased expression of proapoptotic proteins mediated by the induction of endoplasmic reticulum stress with inducing ceramide levels in pancreatic cancer cells is a result of the apoptosis‐facilitating effect of a decrease in fatty acid levels [96]. Malonyl‐CoA accumulation was observed when FAS activity was inhibited, which is known to inhibit CPT‐1 activity. As a consequence, any free fatty acids are likely to be condensed to 3‐keto dihydrosphingosine and then to various ceramides via a series of reduction and acylation steps [93, 97]. According to one study, the elevation of the ceramide level following fatty acid suppression can activate the expression of the proapoptotic protein, BNIP3, in breast cancer cells [98]. As a result, BNIP3 has been suggested as an important mediator of apoptosis induction following the EGCG‐induced suppression of the DNL pathway in cancer cells. The relevant results showed that increasing in the expression of BNIP3 following the treatment of hepatoma cells with concanavalin A inhibited the expression of Bcl‐2/Bcl‐2‐associated X protein ratio, resulting in mitochondrial dysfunction and the activation of caspase‐dependent apoptosis. A decrease in Bcl‐2 expression was found to trigger a translocation of Bak from cytosol to the mitochondria, resulting in the promotion of apoptosis [28].

In addition to the enhanced proapoptotic expression that resulted from fatty acid suppression, a reduction in FAO has been reported as a consequence of the suppression of the ACC‐α expression in hepatocellular carcinoma cells. Both glycolysis and OXPHOS were suppressed by the knockdown of ACC‐α, leading to a decrease in tumor growth [31]. Mitochondrial FAO generates NADH, FADH_2_ and acetyl CoA in each round by shortening fatty acids (two carbons per cycle) to enter the electron transport chain for ATP production [99, 100]. CPT‐1 is known to catalyze the rate‐limiting step of the FAO process by transferring an acyl group of a long‐chain fatty acid‐CoA to carnitine, resulting in acylcarnitine synthesis, with CPT‐2 activity finally triggering β‐oxidation. The downregulation of CPT‐1A in ovarian cancer cells was found to decrease FAO and ATP production [101]. In addition, the inhibition of FAO by etomoxir, an irreversible CPT‐1 inhibitor, promotes apoptosis [99] through the suppression of ATP production in cancer cells [102]. Moreover, the inhibition of FAO by the inner mitochondrial membrane acylcarnitine carrier caused apoptosis in glioblastoma. This finding suggested an important correlation between the CPT‐1 and FAO activity and cancer cell survival [103]. A similar finding was reported in our previous study, which found that EGCG inhibited CPT‐1 activity in HepG2 cells, resulting in ATP production suppression and apoptosis [27]. Therefore, consistent with previous findings, the present results could suggest that the EGCG‐induced inhibition of fatty acid synthesis contributes to the suppression of FAO, leading to a reduction of ATP production that finally induces apoptosis in colon cancer cells. However, the effect of EGCG on FAO‐ATP suppression mediated by the inhibition of CPT‐1 activity was not evaluated in the present study; this is a limitation of the study that necessitates additional research.

In addition to the *in vitro* study, EGCG administration in tumor xenografts of mice did not result in weight loss in the current investigation. According to a previous study using *in vivo* A549 lung cancer xenografts demonstrated that EGCG, in contrast to C75, exerted no weight loss‐related effect on CPT‐1 activity [104]. EGCG treatment in tumor xenografts of mice did not cause weight loss, despite having no effect on CPT‐1 activity in SK‐Br‐3 human breast cancer cells, which was consistent with the results observed in an *in vitro* model [30]. Thus, the effect of EGCG on CPT‐1 activity in animal xenografts requires further evaluation.

It has been reported that the antioxidant system of glioblastoma cancer cells was found to inhibit fatty acid oxidation‐induced apoptosis by increasing ROS‐induced oxidative stress and depletion of nicotinamide adenine dinucleotide phosphate‐reduced glutathione levels [100]. The elevation of ROS levels critically disturbed mitochondria homeostasis, causing a decrease in mitochondrial membrane potential, and, as a result, a decrease in mitochondrial ATP production [105]. In the present study, the depletion of ATP content was linked to an EGCG‐induced decrease in MMP, indicating an impairment in mitochondrial function. This result suggests the correlation of fatty acid metabolism and mitochondrial ATP generation following EGCG treatment.

In cancer cells, EGCG suppressed receptor tyrosine kinase activity, inhibiting cell proliferation. This included epidermal growth factor receptor (also known as erbB1) and human epidermal growth factor receptor‐2, as well as neu/erbB2. The inhibition of these protein targets resulted in the inhibition of downstream signaling cascades, leading to induction of apoptosis in colon cancer cells [106]. EGCG has been shown in several studies to have anticancer effects by directly inactivating intracellular signaling cascades, including as ERK and Akt. The MAPK pathway, which belongs to the serine/threonine kinase family, has been linked to cancer cells apoptosis regulation. This signaling is connected to a downstream PI3K/Akt/mTOR pathway, which plays a key role in controlling cancer cell proliferation and migration. In response to EGCG treatment, the observed reduction in ERK1/2 phosphorylation in colon, pancreatic and lung cancer cells was found to promote the expression of the Bcl‐2 family of apoptotic proteins [107]. ProEGCG, a derivative of EGCG, significantly reduced the phosphorylation of ERK and Akt in endometrial cancer cells, suggesting that the PI3K/Akt/mTOR and MAPK signaling pathways play an important function in the apoptotic pathway [108]. In addition, an inhibitory phosphorylation of the Akt signaling pathway has been discovered to be a potential EGCG mechanism underlying the induction of apoptosis in HCC and colon cancer cells [45, 47]. Furthermore, phosphorylation of the ERK1/2 and PI3K/Akt pathways by ECG and EGCG dimer treatment has been found to inhibit cell proliferation in CRC Caco‐2 cells [4]. Following EGCG treatment, phosphorylation of Akt and ERK in the present study was suppressed, suggesting that EGCG‐induced apoptotic cell death is mediated by MAPK and ERK, which are related to the Akt signaling pathway. Therefore, the present findings regarding EGCG treatment supported the suggestion that the apoptotic effect of EGCG in CRC cells was potentially triggered by an upstream cellular signaling cascade pathway involving MAPK and ERK1/2.

Of note, in addition to regulating apoptosis, the activation of Akt and ERK1/2 pathways has been found to regulate fatty acid synthesis in the DNL pathway. The induction of apoptosis in SK‐Br3 breast cancer cells has been reported to involve a suppression of the DNL pathway, which is controlled by the phosphorylated PI3K/Akt and MAPK/ERK1/2 pathways [109]. The inhibition of FASN by the gallate derivatives of EGCG, which induced apoptosis in human breast cancer cells, has also been suggested to occur under the blocking of AKT and ERK1/2 phosphorylation [110]. Similarly, the anti‐tumor effect of EGCG also occurred in parallel with a decrease in FASN levels, which was found to be associated with a decreased level of AKT and ERK1/2 phosphorylation in triple‐negative breast cancer [79]. Therefore, the apoptotic effect of EGCG, which was regulated by fatty acid depletion, might be triggered by an upstream cellular signaling cascade pathway, p‐AKT and p‐ERK1/2, in CRC cells. However, FASN has also been reported to be an upstream regulator of AKT phosphorylation. Yellen and Foster [111] suggested a feedback activation of Akt and ERK in K‐Ras‐driven cancer cells in response to FASN inhibition. By contrast, depletion of fatty acid level by knocking down of FASN in bladder cancer cells has been reported to suppress p‐AKT protein expression. This could be a result of a disruption in membrane phospholipid synthesis, which is considered to be a modulator of AKT activation [112]. Therefore, following the inhibition of lipogenesis, there would be no fatty acid precursors available for phospholipid synthesis to maintain the activity of membrane platforms for cell signaling. The suppression of p‐Akt and p‐ERK has also been found to downregulate ATP generation from glucose metabolism, resulting in antiproliferative and anticancer effects in non‐small cell lung cancer [113]. The results of the present study suggested that the apoptotic effect of EGCG in CRC cells, which is mediated by the suppression of fatty acid and ATP synthesis, is regulated by the activity of Akt and ERK pathways.

In cancer cells, a reduction in ATP that results in an increase in ADP or AMP levels has been shown to activate AMPK. Glycogen, fatty acid and protein synthesis have all been demonstrated to be inhibited by active AMPK. AMPK activation has been found to suppress FASN activity in hepatoma cells [37, 114], as well as inhibit ACC activity by enhancing phosphorylated ACC levels [82, 115]. In line with previous reports, it is reasonable to assume that, following the inhibition of fatty acid synthesis by EGCG, there may be a positive correlation between ATP levels and the activities of proteins involved in the fatty acid synthesis pathway in colon cancer cells. ATP has also been reported to be a phosphate donor for protein kinase activity, with ATP depletion suppressing ERK activation in thyroid cancer cells [116].

In cancer cells, EGCG was reported to reduce FASN activity and free fatty acid levels without affecting protein expression [27, 117, 118]. EGCG also reduced FASN and free fatty acid levels in lung cancer cells at the same time as inhibiting fatty acid synthesis without affecting protein expression in lung cancer xenograft tumors. It was speculated that, as described for C75, its activity was inhibited without changing protein levels, and it promoted apoptosis in cancer cells by inhibiting fatty acid synthesis at the same time as having no effect on FASN protein abundance [104]. Similarly, EGCG reduced FASN activity in the SK‐Br‐3 cells at the same time as having no effect on the abundance of FASN protein expression [30]. In addition, a previous study demonstrated that an increase in p‐AMPK caused by EGCG contributes to the inhibition of the activities of enzymes involved in fatty acid biosynthesis and lipid accumulation, both of which are involved in cancer growth [114]. Consistent with those results, it was found in the present study that the expression of DNL proteins in xenograft mouse tumors was not affected after 7 days of EGCG treatment. This is the limitation of our study. Further investigations are required to fully comprehend this evidence. By n contrast to the *in vitro* results, G28UCM, a synthetic FASN inhibitor, was found to induce apoptosis in breast carcinoma xenografts by decreasing FASN enzymatic activity but not total FASN levels. However, the mechanism underlying this was not addressed [119].

In the *in vivo* model, we hypothesized that there may be an additional signaling mechanism and time‐dependent protein expression that regulate protein expression in fatty acid synthesis. In the present *in vivo* experiment, the phosphorylation of Akt and ERK was inhibited, although there was no alteration in the abundant expression of PI3K, mTOR and SREBP‐1c following EGCG treatment. This effect is more likely because the concentration of EGCG in the body varies over time. The phosphorylation of MAPK, c‐Jun N‐terminal kinase (JNK) and ERK in HT‐29 cells suggests that EGCG treatment affected signaling cascade in a time‐ and dose‐dependent manner. JNK activation occurred prior to an increase in ERK activation, and was followed by the inactivation of JNK during the late stages of cell death. In addition, ERK activation required a lower EGCG concentration than that of JNK. In response to cellular stress, the evidence revealed interactions among upstream signaling pathways that cross‐talk with one another [120]. Shimizu *et al*. [106] reported a similar occurrence involving signaling molecules following EGCG treatment in human CRC cells. Following the absence of any alterations during the first period of the treatment, ERK phosphorylation was found to be significantly decreased. In a mouse model, a haw pectin produced from hawthorn fruit administration reduced fatty acid synthesis in the liver by downregulating the expression of DNL enzymes after 10 weeks rather than 4 weeks, implying that the inhibitory effect on DNL enzyme expression is time‐dependent [121].

The protein expression pattern of 5FU treatment in the present study was similar to that of EGCG treatment, which was consistent with a previous study showing that the changes caused by 5FU injection in an orthotopic xenograft of human gastric cancer cells in the gastric submucosal layer from nude mice on the protein expression of the PI3K/Akt/mTOR pathway were small [122]. Furthermore, our results suggested that EGCG could be used as anticancer agent without creating undesirable side effects for healthy organs of the tumor‐bearing host.

## Conclusions

The results of the present study demonstrated that EGCG inhibited fatty acid synthesis, resulting in the depletion of ATP, a regulator of the of fatty acid synthesis, and hence contributed to the induction of apoptosis in colon cancer cells, both *in vitro* and *in vivo*. This study provided the basis for further research into using EGCG to target the DNL pathway as a potential clinical approach for the treatment of CRC cells. To fully understand the signaling pathways that govern the DNL pathway in response to EGCG therapy, further research is required.

## Conflict of interest

The authors declare no conflict of interest.

## Author contributions

PK and PS were responsible for study conceptualization, data curation and formal analysis. PS was responsible for funding acquisition. PK, DS, MS, DP, SP, JS and PS were responsible for the conducting the investigation. PK, JS and PS were responsible for methodology. DS, MS, DP, SP, JS and PS were responsible for project administration. PK, DS, MS, LR, DP, SP, JS and PS were responsible for writing, review and editing.

## Data Availability

The data that support the findings of the present study are available within the article.
